# Bacteriological Identification, Characterization and Changes of Feces Microbiome in Prostate Cancer Patients Undergoing Radiotherapy

**DOI:** 10.2147/CMAR.S517416

**Published:** 2025-08-29

**Authors:** Aleksandra Bogumiła Florkiewicz, Paweł Fijałkowski, Piotr Fijałkowski, Michał Złoch, Agnieszka Ludwiczak, Dorota Gabryś, Wioletta Miśta, Jolanta Mrochem-Kwarciak, Anna Jędrzejewska, Ewa Telka, Małgorzata Rabsztyn, Grażyna Czeszewska-Rosiak, Radik Mametov, Andrzej Tretyn, Paweł Pomastowski

**Affiliations:** 1 Centre for Modern Interdisciplinary Technologies, Nicolaus Copernicus University in Torun, Torun, Poland; 2 Radiotherapy Department, Maria Sklodowska-Curie National Research Institute of Oncology, Gliwice Branch, Gliwice, Poland; 3 Analytics and Clinical Biochemistry Department, Maria Sklodowska-Curie National Research Institute of Oncology, Gliwice Branch, Gliwice, Poland

**Keywords:** fecal, microbiome, microorganisms, matrix-assisted laser desorption/ionization time-of-flight, prostate cancer

## Abstract

**Purpose:**

To investigate the bacteriological characteristics of the gut microbiome in patients with prostate cancer and changes during and after radiation therapy.

**Patients and Methods:**

Forty-one prostate cancer (PCa) patients treated with radiation therapy were included in the study. Fecal samples were collected at three points: before gold marker implantation into the prostate gland (K1), at the start (K2), and last day of radiotherapy (K3). Microbial identification was performed using MALDI-TOF MS, which allowed for precise identification at the species and genus levels. Blood biochemical parameters were assessed, and correlation analyses were performed.

**Results:**

In total, 291 microbial isolates were identified, with the most common genera being *Escherichia* (N=120), *Streptococcus* (N=31), and *Enterococcus* (N=30). A significant decrease in *E. coli* was observed in K3 compared with K1 and K2, whereas *Citrobacter* appeared exclusively at K2. Additionally, liver enzyme levels decreased, and IL-6 levels increased during treatment. These findings indicate significant shifts in the gut microbiota due to radiotherapy.

**Conclusion:**

Radiation therapy alters the gut microbiota composition in patients with PCa, reduces microbial diversity, and promotes the growth of opportunistic pathogens. These changes are linked to biochemical parameters, suggesting a potential impact on health. Further research is needed to explore microbiome-targeted interventions during treatment.

## Introduction

A complex trillion microbial communities of bacteria, protozoa, fungi, and viruses together shape the humane gut microbiota; 99% of all microorganisms in the human body are located in the digestive tract.^1^ It is no coincidence that the gut is referred to as the “second brain”; it is a habitat for microbes that are crucial not only locally but also a systemic level, such as in the case of the gut-brain axis.^2^,^3^ Bacteria in the digestive tract can be divided into three principal groups based on their effect on host health: pathogenic, potentially harmful, and beneficial. Pathogenic bacteria can cause food intolerance, infections, and inflammation. Potentially pathogenic species, such as *Escherichia coli* or *Enterococcus* species, are part of the normal microflora under regular conditions, but their excess can lead to the aforementioned undesirable inflammatory states and numerous serious diseases, including cancer.^4^ The gut microbiota can therefore be involved in all stages of cancer, including initiation, progression, treatment outcomes, and side effects.^5–9^ The presence of a tumor may increase the diversity of microorganisms at the tumor site, which may lead to a decrease in gut microbiota diversity, potentially serving as a precursor to the onset and development of cancer, and even directly contributing to its formation and progression.^10^

Prostate cancer (PCa) is the most common cancer in men in Europe, with a declining mortality rate owing to advancements in treatment. Interestingly, among Eastern European countries, Poland was the only country with an increasing mortality trend due to PCa.^11^,^12^ Unfortunately, the impact of the gut microbiome microenvironment on the development and progression of PCa is closely linked to the risks of obesity and inflammation, which can directly affect organs like the prostate.^13^ Carcinogenicity can also be induced by metabolites from gut microorganisms, such as fatty acids and polyamines, that act on distant organs.^14^

Equally significant in the biology of prostate cancer are microRNAs (miRNAs) – small, non-coding RNA molecules that regulate gene expression at the post-transcriptional.^15^ Aberrant expression of specific miRNAs has been implicated in the pathogenesis and progression of numerous cancers, including prostate cancer. Certain miRNAs have been shown to function as oncogenes, promoting cancer cell proliferation, migration, and invasion, while others act as tumor suppressors, inhibiting these processes.^16^ Current therapeutic strategies are actively investigating the potential of targeted delivery of miRNAs (both miRNA mimics and antagomirs) to prostate cancer cells. Advances in nanotechnology, exosome utilization, and ligand-receptor conjugates are opening novel avenues for enhancing the specificity and efficacy of this therapy, while minimizing off-target effects.^17–19^ The therapeutic potential of miRNAs in prostate cancer is promising, and ongoing research is focused on overcoming challenges related to the stability, delivery, and in vivo biodistribution of these molecules. The fundamental importance of microRNAs was recognized in 2024, when Ambrose and Ruvkun were awarded the Nobel Prize in Physiology or Medicine for their discovery of microRNAs and their role in post-transcriptional gene regulation, which initiated extensive research into the role of miRNAs in various biological processes and diseases.^20^,^21^ The literature presents speculations and preliminary evidence suggesting the existence of complex interactions between the gut microbiome and miRNAs in the context of various diseases, including cancers. The mechanisms underlying these interactions are not yet fully elucidated, but they encompass the potential modulation of miRNA expression by bacterial metabolites or bacterial cell wall components. Conversely, miRNAs secreted by host cells may influence the composition and function of the gut microbiome.^22–25^

The pathophysiology of radiation-induced bowel injury is derived from a complex interplay between epithelial injury and alterations in the enteric immune, nervous, and vascular systems in genetically predisposed individuals. Moreover, gut microorganisms can cause intestinal inflammation and play an important role in the development of radiation-induced bowel injury.^26^ Risk factors include radiation dose and combined therapy use, with symptoms including diarrhea, abdominal pain, nausea, and vomiting. Studies on the gut microbiome of patients with PCa have shown an increased presence of bacteria such as *Roseburia, Clostridium* cluster *IV* (now family *Ruminococcaceae*), and *Faecalibacterium* in patients with symptoms of radiation-induced enteropathy.^27^

In recent years, there has been rapid development in the use of 16S rRNA bacterial genome sequencing and shotgun sequencing as analytical tools for studying the human microbiome.^28^ It is predicted that future cancer treatments, particularly for PCa, will involve the microbiome, as it plays a significant role in immune system regulation. Therefore, rapid and accurate identification of microorganisms, including pathogenic pathogens or microbes involved in inflammation during carcinogenesis, is essential to enable early and effective treatment.^29^,^30^ The major limitation of the genome-based analysis of the microbiome, such as the analysis of 16S amplicons, is that it mainly refers to dominant microbiota omitting the minor populations and rare species, generally enables identification only at the genus level as well as does not provide information about cells status, namely, if the bacteria are live or death.^31^ The modern culturomics approach, which consists of multiple culture conditions for microbial isolations combined with rapid isolate identification via the matrix-assisted laser desorption/ionization time-of-flight (MALDI-TOF MS) technique, may fulfill metagenomic gap, providing new light on changes in the gut microbiota during cancer treatment. In this study, we performed microbial identification analyses of stool samples from PCa patients before, during, and after radiotherapy using MALDI identification, which is increasingly being used in microbiological laboratories and clinical diagnostics.^32^,^33^

It has become clear that the fecal microbiome is associated with PCa-related risk factors, and that gut bacteria can produce metabolites that influence this type of malignancy. However, much remains unknown about their role and impact on PCa.^13^ The changes in the fecal microbiome during treatment, particularly regarding the presence or absence of certain microorganisms in response to radiation, remain unclear. Moreover, although the prostate does not have direct contact with lower sections of the gastrointestinal tract, including the colon, the role of the gut microbiota in this process is still under investigation. In our study, we focused on investigating the effect of PCa radiotherapy on fecal microbiome changes using MALDI in a culturomic approach.

## Materials and Methods

### Characteristics of Patients and Patient Recruitment

The study included patients who underwent radiation therapy primarily for PCa with or without hormonal treatment. Before starting radiation therapy planning, one to three gold markers were implanted in each patient. Radiation therapy planning, radiation treatment, and post-treatment follow-up were conducted at a single center of the Maria Skłodowska-Curie National Cancer Institute (Gliwice Branch) in accordance with the established protocol. Forty-one patients with different PCa stages were recruited for this study.

Patient characteristics are shown in Table 1.

**Table 1:** Patient Characteristics

Patient Characteristics	N/%
Age median 67.2 (range 46–80)	
**Clinical stage/Timepoints**	
K1	41/33.3%
K2	41/33.3%
K3	41/33.3%
PSA	
<10	22/48.9%
10–20	15/33.3%
>20	8/17.8%
Gleason score	
6	15/33.3%
7 (3 + 4)	6/13.3%
7 (4 + 3)	10/22.2%
8	8/17.8%
9–10	6/13.3%
Use of androgen deprivation therapy	
Yes	35/77.8%
No	10/22.2%
Treatment	
Fractionated Linear accelerator	27/60%
SBRT Linear accelerator	3/6.7%
SBRT Cyberknife	15/33.3%
Irradiated volume	
Prostate alone	28/62.2%
Prostate bed + pelvic lymph nodes	17/37.8%
Prostate + pelvic lymph nodes	1/2.2%

**Abbreviations**: PSA, Prostate Specific Antigen; SBRT, Stereotactic Body Radiation Therapy.

#### Choice of Radiotherapy Method, Volume, and Dose of Gold Marker Implantation

The choice of radiotherapy modality, volume, and individual gold tracer implantation doses in patients with PCa were described in our earlier paper.^34^ Participation in the study did not influence the choice of treatment method, irradiated volumes, or dosage. The patients received hormonal therapy with LH-RH analogs with or without flutamide. Radiotherapy was administered using a linear accelerator or CyberKnife. The irradiated volumes included the prostate, the prostate with the base of the seminal vesicles, or the prostate with seminal vesicles, with a total dose of 76–78 Gy delivered in 2 Gy per fraction, or hypofractionation of 36.25 Gy delivered in 7.25 Gy per fraction, using MV photons. Two patients received an additional 15 Gy boost to the prostate using brachytherapy. If necessary, pelvic lymph nodes were irradiated with a total dose of 44–50 Gy in 2 Gy per fraction, with an optional boost to the affected lymph nodes to a total dose of 60–68 Gy or delivered as a stereotactic boost of 16 Gy in 2 fractions. All necessary approvals, including ethical ones, were obtained. The study was approved by the NIO-PIB Ethics Committee KB/430-104/19 and conducted in accordance with Good Clinical Practice principles. Written informed consent was obtained from all the patients.

Men who were initially or postoperatively radically irradiated for PCa were included in the study. The treatment was performed at the Maria Sklodowska-Curie National Institute of Oncology, National Research Institute, Gliwice Branch, according to the manufacturer’s protocol. Three stool samples were collected for examination before gold marker implantation (the tracer was implanted into the prostate gland for precise irradiation) (K1), at the start (K2), and on the last day of radiotherapy (K3). Antibiotic therapy was administered before gold fiducial implantation; therefore, azithromycin (AZM, 500 mg) or ciprofloxacin (CIP, 250 mg) was usually prescribed. Enema infusions are usually prescribed to clean the rectum prior to implantation.

#### Blood, Urine, and Fecal Sample Collection

Blood and urine samples were used for routine biochemical tests, and one urine sample was used for general examination and urine culture, which were routinely performed during RT. The course of urine and blood sampling was described in our previous work.^35^ The stool samples were collected from the patients into sterile containers for MALDI-TOF MS analysis. The fecal samples collected were characterized by all possible types according to The Bristol Stool Form Scale. After sampling, the stool container was frozen at −80°C and transported on dry ice to the Centre for Modern Interdisciplinary Technologies of Nicolaus Copernicus University in Torun for microbial identification analysis using MALDI-TOF MS.

#### Blood and Urine Parameters

Routine tests were conducted on the blood samples at various time points. These tests included blood morphology, liver function, lipid profile, creatinine, electrolyte, glucose, C-reactive protein (CRP), interleukin 6 (IL-6), and PSA levels. Urine samples were subjected to general tests, including color, transparency, pH, specific gravity, and standard urine cultures, which were mandatory for each patient during radiotherapy. An anaerobic medium was also used.

Detailed information about patients’ characteristics was included in the dataset stored in the RepOD (see *Data Sharing Statement*).

### Isolation and Culturing of Bacteria from Fecal

For microbiological culture, fecal samples were thawed at room temperature, after which appropriate dilutions were prepared. One gram of each stool sample was added to 9 mL of sterile buffered peptone water (Sigma Aldrich, Steinheim, Germany) to obtain dilutions of 10^−5^ and 10^−6^, and diluted stool samples (100 µL) were collected and inoculated on Petri dishes (Alchem, Torun, Poland) with the appropriate culture medium. Different types of culture media were used: Schedler agar, *Lactobacillus* agar according to DeMan, Rogosa, and Sharpe, Mueller Hinton Agar, Bromocresol Purple Lactose Agar (Sigma Aldrich, Steinheim, Germany), and Columbia Blood Agar (Oxoid, Basingstoke, Great Britain). BLA medium was prepared by adding defibrinated sheep blood (GRASO Biotech, Poland) to a final concentration of 5% (*v/v*).

The bacteria were then incubated at 37°C for 24–48 h. To obtain pure cultures, single colonies with different morphologies were transferred to new Petri dishes, inoculated, and incubated at 37°C for 24 h.

### Microbiome Analysis Using Matrix-Assisted Laser Desorption Ionization Time of Flight MS Technique

The MALDI-TOF MS technique, MALDI Biotyper 3.0, (Bruker Daltonics GmbH, Bremen, Germany), and a bacterial protein extraction protocol according to Bruker guidelines were used to identify microorganisms, as described in an earlier paper by our team.^36^

The samples were analyzed using an UltrafleXtreme MALDI-TOF mass spectrometer (Bruker Daltonics GmbH, Bremen, Germany) equipped with a smartbeam-II laser-positive mode. Spectra were collected manually using the manufacturer’s flexControl software, using the parameters described in a previous paper.^37^ Collected spectra were subjected to Savitzky-Golay smoothing, baseline correction with the TopHat algorithm, and calibration using a Bruker bacterial test standard (Bruker Daltonics GmbH, Bremen, Germany) in quadratic mode using the manufacturer’s flexAnalysis software. Each sample was measured in duplicate.

### Statistical Analysis

Data analysis and chart generation were conducted using the PS IMAGO PRO 9.0 package (version 29.0.0.0, Predictive Solutions, Poland). Additionally, the Python environment (version 3.8) was employed with the Pandas library (version 1.2.0) and Matplotlib (version 3.4.0).

The Shapiro–Wilk test was used to evaluate the normality of numerical variables. To assess the homogeneity of variances, Levene’s test was applied. Fisher’s exact test was applied for pairwise comparisons to assess significant differences in levels of identification, the number of genus and species between K1, K2, and K3. Cluster analysis was performed to demonstrate the distribution of species in each cluster over the different time points. The Friedman test was employed to examine differences in numeric variables across the three time points (K1, K2, K3). To determine which specific pairs of time points differed significantly, post hoc Wilcoxon signed-rank tests were conducted with Bonferroni correction to adjust for multiple comparisons and control the family-wise error rate. For categorical data, associations between bacterial species and sampling time points were analyzed using the Chi-square test of independence. To quantify the strength of these associations, Cramér’s V was calculated. Statistical significance was set at p ≤ 0.05. Results from post hoc analyses with Bonferroni-adjusted p-values ≤ 0.01 were considered highly significant.

## Results

### MALDI Identification Levels of Faecal Samples

**Figure 1:**
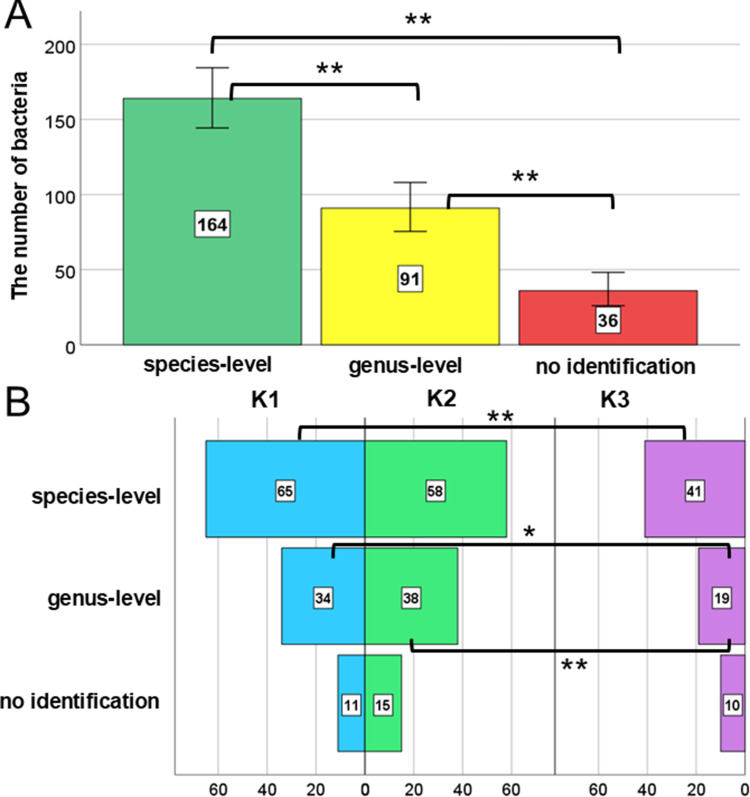
Distribution of bacterial isolates identified by the MALDI system at the species, genus, and unclassified levels (**A**), with identification levels categorized by the following time points: before gold marker implantation into the prostate gland (K1), at the start (K2), and last day of radiotherapy (K3) (**B**). Error bars represent 95% confidence intervals. Levels of statistical significance were indicated by asterisks: *p ≥ 0.05, **p ≥ 0.01.

A total of 291 isolates were submitted for MALDI identification, of which 56% (N = 164) were identified at the species level, 31% at the genus level (N = 91), and 12% remained unidentified (N = 36) (Figure 1A), with statistically significant differences observed between the three identification levels. The identification levels varied depending on the time points of radiotherapy duration (Figure 1B). The highest number of species-level identifications was observed at time point K1 (N = 65), with no statistically significant differences between K1 and K2 (*p* = 0.494) or between K2 and K3 (*p* = 0.054). Fisher’s test indicated a significantly higher number of species-level identifications at K1 than at K3 (*p* = 0.006). Statistically significant differences in genus-level identification were observed between K1 and K3 (*p* = 0.022) and between K2 and K3 (*p* = 0.004). No statistically significant differences in the “no identification” levels were found between K1, K2, and K3.

### Bacterial Composition of Fecal Samples

**Figure 2:**
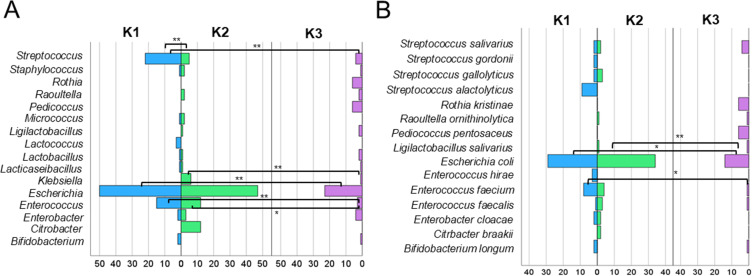
Genus distribution of identified isolates at different time points relative to radiotherapy (K1, K2, K3) (**A**) with species distribution of identified isolates at the same time points (**B**) determined by the MALDI system. The 1% most frequently occurring genera and species were shown. Levels of statistical significance between K1, K2, and K3 were indicated by asterisks: *p ≥ 0.05, **p ≥ 0.01.

The most frequently identified bacterial genera in the fecal samples were *Escherichia* (N = 120), *Streptococcus* (N = 31), and *Enterococcus* (N = 30) (Figure 2A). Statistically significant differences in the frequencies of these genera were observed depending on the time point after RT. Significantly more *Escherichia* were identified at K1 than at K3 (*p* = 0.00024) and between K2 and K3 (*p* = 0.00101). Significantly more bacteria were classified for *Streptococcus* at K1 compared to K2 (*p* = 2.64 × 10^−^^5^) and K1 compared to K3 (*p* = 6.40 × 10^−^^6^). A significantly higher number of *Enterococcus* bacteria was noted at K1 than at K3 (*p* = 0.0015) and K2 than at K3 (*p* = 0.0153). Additionally, the genera *Rothia, Pediococcus*, and *Candida* were absent in K1 and K2 samples; however, they were present in K3, whereas *Citrobacter* was only detected in samples from K2. The genus *Escherichia* was represented by a single species, *E. coli* (N = 77) (Figure 2B). In contrast, *Streptococcus* was represented by four different species, with *S. salivarius* being the most abundant (N = 8), whereas *Enterococcus* was represented by three species, with *E. faecium* being the most common (N = 13). The number of identified species varied among time points K1, K2, and K3. Statistically significant differences were observed in the number of identified *E. coli* between K1 and K3 (*p* = 0.011), as well as between K2 and K3 (*p* = 0.00085) as well in the number of *Enterococcus faecium* between K1 and K3 (*p* = 0.011) (Figure 2B).

### Species Occurrence in K1, K2 and K3 Time Points

**Figure 3:**
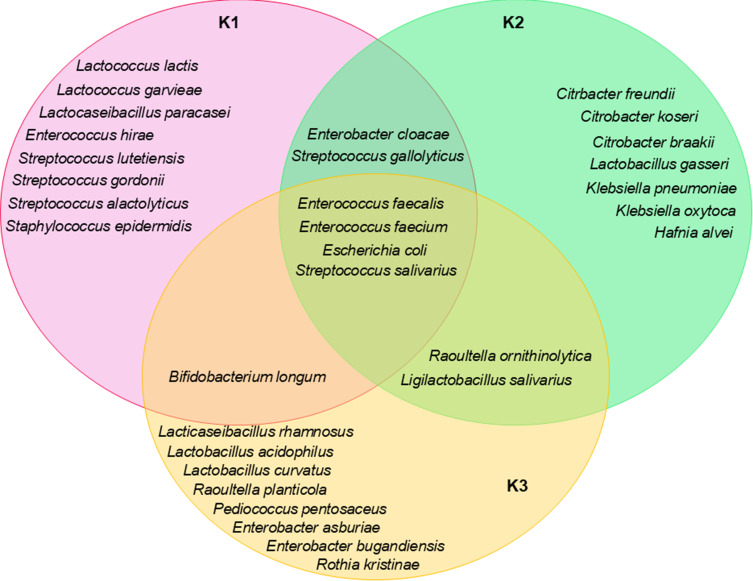
The relationship between the occurrence of specific species at different time interval (K1, K2, K3). Species differentiating between time points, as well as those shared across combinations of K1, K2, and K3 were indicated.

At each time point, unique differentiating bacteria as well as those common across specific time intervals were identified (Figure 3). In the K1 group, eight unique species were identified: *Lactococcus lactis, L. garvieae, Lacticaseibacillus paracasei, Streptococcus alactolyticus, S. lutetiensis, S. gordonii, Enterococcus hirae, and Staphylococcus epidermidis*. In the K2 group, seven species were identified, including *Citrobacter braakii, C. freundii, C. koseri, Klebsiella pneumoniae, K. oxytoca, Hafnia alvei*, and *Lactobacillus gasseri*. Meanwhile, in the K3 group, *Rothia kristinae, Raoultella planticola, Enterobacter asburiae, E. bugandensis, Pediococcus pentosaceus, L. rhamnosus, L. curvatus*, and *L. acidophilus* were identified. Some bacteria were detected in both K1 and K2 (*S. gallolyticus, E. cloacae*), whereas others were shared between K2 and K3 (*R. ornithinolytica, Ligilactobacillus salivarius*). Only one species, *Bifidobacterium longum*, was identified in K1 and K3. Interestingly, four species were consistently identified across all time points: *Escherichia coli, E. faecalis, E. faecium*, and *S. salivarius*.

A statistically significant relationship between the bacterial species and the time of isolation (K1, K2, K3) was confirmed by the chi-square test, with a chi-square statistic of approximately 116.01 (p ≤ 0.01). In addition, Cramér’s V coefficient was used as a measure of the strength of the relationship. The Cramér’s V (V = 0.595) confirmed a strong association between certain bacterial species and specific time points. *Escherichia coli* exhibited a marked decrease in frequency at time point K3, *S. alactolyticus* demonstrated a strong association with time point K1; *R. kristinae* and *P. pentosaceus* were found exclusively at time point K3, and *Enterococcus faecium* showed a significant decline in prevalence from K1 to K3.

**Figure 4:**
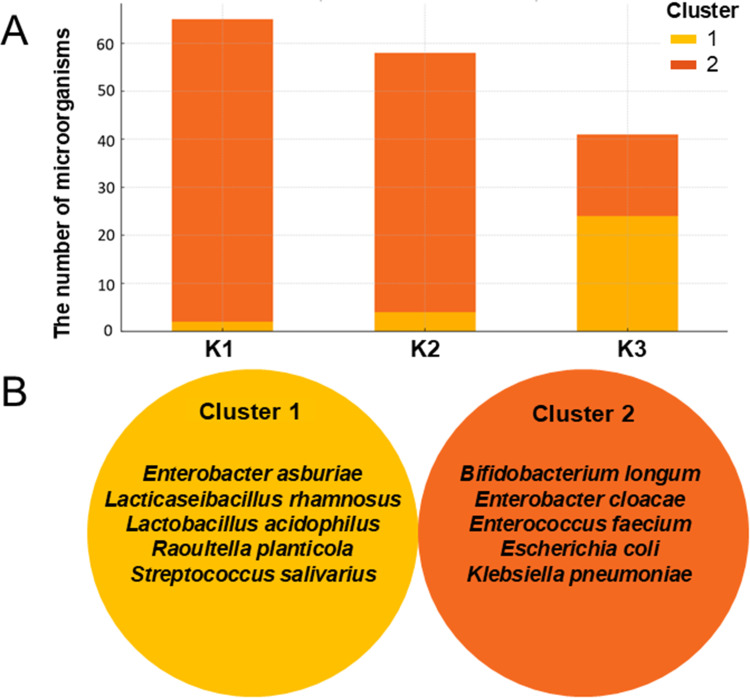
The distribution of the number of microorganisms in two clusters during the different stages of radiotherapy (**A**) with the identification of dominant species in each cluster (**B**).

A temporal-dependent shift in the number and type of microorganisms was observed during radiotherapy progression (Figure 4A). A predominance of Gram-positive bacteria was noted in Cluster 1, in contrast to Cluster 2, where Gram-negative bacteria were dominant. In Cluster 1, a low number of microorganisms was observed at K1 and K2, followed by a significant increase at K3. Conversely, Cluster 2 showed a high count of microorganisms in K1, which gradually decreased at K2 with a sharp decline observed in K3. Over time, the species from cluster 1 appeared to have replaced those from cluster 2. The dominant species in Cluster 1 were primarily identified in K3 Time, including *E. asburiae, L. rhamnosus, L. acidophilus, R. planticola*, and *S. salivarius*. In contrast, the dominant species in cluster 2, such as *B. longum, E. cloacae, E. faecium, E. coli, and K. pneumoniae* were more prevalent before radiotherapy (K1 and K2) (Figure 4B).

A comparison of the MS spectra of the species identified at each time point showed changes in the position of the signals, which may be indicative of changes occurring at the proteome level. The best method for visualizing these changes is the GelView tool (Supplementary Materials Figure 1).

### Biochemistry of Blood Parameters Over the Radiotherapy

**Figure 5:**
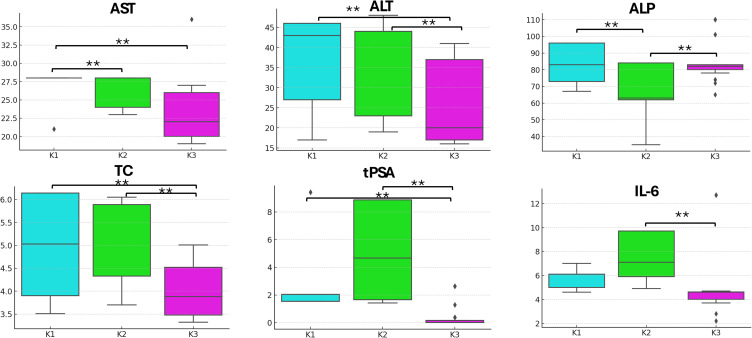
The biochemical parameters changes across the points K1, K2, and K3. Levels of statistical significance between time points are indicated by asterisks: *p ≥ 0.05, **p ≥ 0.01.

**Abbreviations**: AST, Aspartate Transferase; ALT, Alanine Transaminase; ALP, Alkaline Phosphatase; TC, Total Cholesterol; tPSA, total Prostate Specific Antigen; IL-6, Interleukin-6.

Statistically significant differences in selected biochemical blood parameters were observed after radiotherapy (Figure 5). A decrease in aspartate transferase (AST) and alanine transaminase (ALT) activities was observed as treatment progressed, with a significant reduction between K1 and K3 (*p* = 0.0019 and 0.0045, respectively) and between K2 and K3 (*p* = 0.001 and 0.0004, respectively). In contrast, alkaline phosphatase (ALP) activity was not significantly different pre- and post-radiotherapy (*p* = 0.9257). Total cholesterol (TC) levels were significantly reduced between K1 and K3 (*p* = 0.0009) and between K2 and K3 (*p* = 0.0). The lowest mean concentration of total prostate-specific antigen (tPSA) was observed at K3, which was significantly lower than that at both K1 and K2 (*p* = 0.0). Additionally, interleukin-6 (IL-6) levels were significantly lower in K3 and K2 groups (*p* = 0.0241).

### Correlation Between Fecal Microbiome, Urine and Blood Parameters

**Figure 6:**
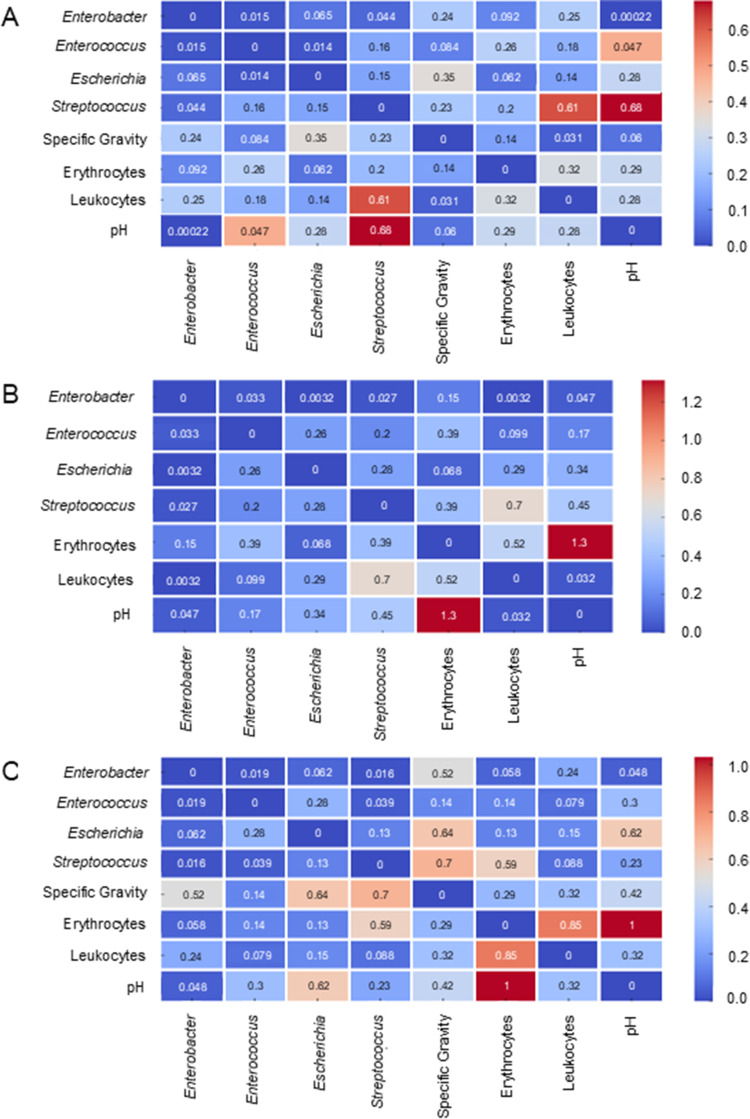
Absolute differences in correlation strengths between the most represented bacterial genera and urine parameters between the respective time points (K1, K2, and K3). (**A**) Differences between K1 and K2, (**B**) differences between K1 and K3, and (**C**) differences between K2 and K3 were presented.

A significant difference in the correlation changes between K1 and K2 was demonstrated for the genus *Streptococcus* and urine leukocyte levels (0.61), urine pH (0.68), and *Enterococcus* and pH (0.47). Similar correlations were observed between the presence of the four most common bacterial genera at K1 and K2 (Figure 6A). Significant differences were also observed in the presence of *Streptococcus* and leukocyte levels between K1 and K3 (0.7) (Figure 6B). However, the most pronounced differences in the correlations between bacterial genera and the selected urine parameters were observed between K2 and K3 (Figure 6C). The strongest differences involved *Escherichia* and *Streptococcus* in relation to urine specific gravity (0.64 and 0.7, respectively).

## Discussion

As the largest concentration of bacteria in the human body, the gut microbiota can influence the occurrence and development of cancer by affecting the functioning of the tumor microenvironment, and consequently impact the effectiveness of the undertaken cancer treatment.^13^,^37^ On the other hand, it is known that cancer and its treatment harm the gut microbiota, often leading to an imbalance associated with numerous side effects, such as mucositis, diarrhea, and fatigue, in the case of pelvic irradiation in PCa patients, as a result of radiation-induced gut microbiota imbalance.^38^,^39^ This phenomenon is described in the literature as a bidirectional interaction between the gut microbiome and cancer therapies, where these therapies can disrupt the microbiome (eg, promoting dysbiosis), and these disruptions, in turn, can affect the effectiveness of the therapies.^40^ Knowledge about the overall response of the gut microbiome to radiotherapy and its impact on the patient’s condition during treatment remains limited, as the wide variability of techniques used to characterize the taxonomic distribution of the gut microbiota, along with different types of samples studied, varying doses and durations of radiation exposure, and different time points post-irradiation, pose significant limitations in the cumulative interpretation of results.^41^ The heterogeneity of radiotherapy protocols among patients stands out as a key factor that could have impacted the observed microbiome changes. The varying doses, fractionation schemes, and target volumes employed introduce variables with distinct effects on the gut microbiome. Different radiation doses can result in varied levels of intestinal epithelial cell damage and microflora dysbiosis, while different fractionation schedules might affect the microbiome’s recovery capabilities between exposures. Moreover, the volume of irradiated tissue, which determines the extent of intestinal exposure, can significantly influence the scale and type of alterations in microbiota composition. Our findings align with this understanding, particularly in the context of PCa and radiotherapy. By studying changes in the fecal microbiome of PCa patients undergoing radiotherapy, we observed significant shifts in bacterial composition at different time points during treatment (Figures 1 and 2). These shifts correlate with previously reported disruptions in microbiota diversity, further supporting the idea that cancer therapies, particularly radiation, can have profound effects on the microbiome.

Patients diagnosed with PCa frequently present with prostatitis syndrome. In both clinical studies and practice, AZM has demonstrated efficacy in treating Chlamydia-induced prostatitis and other sexually transmitted genitourinary infections.^42^ Another significant challenge is the formation of microbial biofilms, a dominant feature of nosocomial infections. Given the increasing antibiotic resistance of biofilms, rendering monotherapy ineffective, combined therapy appears crucial for their eradication. Research by Saini et al^43^ showed favorable outcomes for AZM + CIP combined therapy in *Pseudomonas aeruginosa*-induced urinary tract infections (UTIs), attributed to a significant reduction in renal and bladder colonization, inflammatory mediator production, and improved histopathology. The combination of a fluoroquinolone with a macrolide seems beneficial in treating experimental *P. aeruginosa*-induced pyelonephritis compared to antibiotics alone.^43^,^44^ Such a synergistic antibiotics combination is particularly desirable in clinical settings.

It is important to note that the use of AZM + CIP aligns with the protocol for fiducial marker implantation, as documented in our previous research.^34^ The administration of these antibiotics, known for their broad-spectrum antibacterial activity, could have contributed to the depletion of the gut microbiome even before the commencement of radiotherapy. This pre-existing microbial alteration might have subsequently influenced the observed changes in microbiome composition during and after radiotherapy, potentially modifying patient response to treatment and the trajectory of radiation-induced dysbiosis (Figures 1 and 2). Therefore, our analytical results may reflect an interaction between initial antibiotic-induced dysbiosis and radiotherapy-related microbiome stress, which could have either masked or amplified certain effects observed in the analyzed stool samples.

Nevertheless, the most common finding is that gut microbiome diversity decreases after radiotherapy.^45^ Our research and analyses confirm this hypothesis. A total of 291 isolates were sent for MALDI-TOF MS identification, with 56% (N=164) identified at the species level, 31% (N=91) at the genus level, and 12% (N=36) remaining unidentified (Figure 1). He et al^45^ presented a procedure in their study that reduced the identification wait time by 2–3 days. They assessed the performance, effectiveness, and costs of the Biotyper system based on MALDI for the routine identification of bacterial pathogens cultured on selective stool media. From 605 stool samples, 304 colonies were selected and identified, including 22 species of *Salmonella*, 39 species of *Shigella*, three isolates of enterohemorrhagic *E. coli* (EHEC), two isolates of *Yersinia enterocolitica*, two species of *Campylobacter*, and 236 isolates of normal non-pathogenic gut flora. Compared to phenotypic methods, the Biotyper system correctly identified 259 isolates at the genus level, representing an accuracy of 85.2%. For the 68 pathogenic intestinal isolates, the genus-level agreement rate was 38.2%, whereas it was 98.7% for isolates from normal gut flora. Among the normal gut flora, *E. coli* constituted the largest proportion (94 isolates, 39.8%). A study conducted by Shrestha et al^46^ in 135 patients with PCa showed an increased presence of *Anaerococcus lactolyticus, A. obesiensis, Streptococcus anginosus, Varibaculum cambriense, Actinobaculum schaalii*, and *Propionimicrobium lymphophilum*. On the other hand, the team led by Alanee et al assessed the urinary and gut microbiota of 30 men undergoing transrectal prostate biopsy, finding that *Veillonella, Streptococcus*, and *Bacteroides* spp. were significantly abundant in patients with PCa, but none of the species were significantly associated with it.^47^ Liss et al^13^ also reported an increased abundance of *Streptococcus* spp. among patients with PCa compared to the control group. The bacterium *B. longum* positively impacts host health by inhibiting inflammation through immune system balance regulation, increased acetate production, and improved gut barrier function.^27^ Conversely, *E. faecalis* is a natural inhabitant of the human gastrointestinal tract but can become dominant and cause infections when gut homeostasis is disrupted; this bacterium is also a cause of bacterial prostatitis.^47^,^48^ Our results are consistent with the literature, which notes that gut dysbiosis – triggered by radiotherapy – can lead to long-term health implications, including inflammation and an increased risk of opportunistic infections.

Our study revealed a gradual decrease in *E. coli* counts, particularly after the completion of radiotherapy. This trend is noteworthy because *E. coli* is a commensal bacterium crucial for maintaining the integrity of the gut barrier. Its depletion may indicate weakening of the gut barrier and subsequent colonization by potentially pathogenic bacteria such as *R. kristinae* and *E. asburiae*, which became more prevalent after treatment. These changes suggest a potential link between reduced microbial diversity and the emergence of opportunistic infections, particularly in immunocompromised individuals. Our findings also showed that *C. braakii* appeared only during radiotherapy, indicating a possible association with inflammatory conditions triggered by the treatment. Similarly, *Klebsiella* was present on the first day of radiotherapy initiation (K2) and on the day of completion (K3), which may indicate ongoing disease processes and increased risk of infection. The presence of these bacteria may result from an altered balance of the gut microbiota, favoring their growth and potentially leading to inflammation or infections in patients, especially those weakened by treatment.

In contrast, *B. longum*, a probiotic bacterium, was present before and after radiotherapy but was absent during treatment. This suggests that radiotherapy directly affects the number of bacteria, possibly by suppressing beneficial bacteria. Its reappearance on the day of radiotherapy completion indicates possible restoration of the intestinal microbiota, which may play a role in restoring the intestinal barrier and improving intestinal health after treatment (Figures 2 and 3).

In PCa patients, an increased number of *E. coli* was observed in prostate secretions, suggesting that this may contribute to the development of PCa.^49^ It is noted that nearly 90% of *E. coli* strains inhabiting the human gastrointestinal tract are commensal bacteria that rarely cause disease.^50^ As a facultative anaerobe, *E. coli* can reduce oxygen levels in the gut, thus creating conditions for the colonization of other anaerobic bacteria. Moreover, *E. coli* is a gut microorganism that is crucial for maintaining proper mucosal permeability by regulating bile acid metabolism in the human colon.^51^,^52^ Therefore, the observed gradual decrease in the number of *E. coli* may indicate a chain of events triggered by radiotherapy, leading to depletion of the natural microbiota, weakening of the gut barrier, and, consequently, increased colonization by microorganisms from outside the gut environment. Thus, the observed percentage decrease in commensal *E. coli* may have contributed to the increase in species diversity of the gut microbiota, including potentially pathogenic bacteria. In studies by Wheeler and Liss,^53^ *Streptococcus* species were found in increased numbers in patients with PCa compared to those in the control group, which was also confirmed by our analyses. *S. alactolyticus* is of particular interest because it was isolated before fiducial marker implantation and appeared in smaller quantities after radiotherapy. It belongs to the *Streptococcus bovis/Streptococcus equinus* (*SBSEC*) group of non-enterococcal streptococci, which inhabit the gastrointestinal tract of animals and humans and participate in food fermentation.^54^ It has also been noted that the prevalence of this bacterium in the human gastrointestinal tract increases in rural areas, which may be due to contact with animal feces and fermented food products.^55^ In contrast, *S. gallolyticus* bacteria were mainly present in samples from patients before fiducial implantation (K1) and before radiotherapy (K2). This species also belongs to the aforementioned SBSEC group and is one of the few opportunistic bacterial species strongly associated with colorectal cancer.^56^ After the completion of radiotherapy (K3), the commensal bacterium *R. kristinae* was isolated. It can cause opportunistic infections in patients with weakened immunity, including those with cancer.^57^,^58^ Lactic acid bacteria, such as *L. lactis, L. garvieae, L. paracasei, L. gasseri, L. curvatus, L. acidophilus, L. rhamnosus* inhibit tumor growth by regulating angiogenesis and directly inducing apoptosis in cancer cells.^59^ Bacteria *E. cloacae* is part of the normal bacterial flora in humans, which, like most members of the *Enterobacteriaceae* family, can cause opportunistic infections in hospitalized patients and those who are immunocompromised.^60^ In the hospital environment, the genus *Citrobacter* can be a source of many infections in weakened, hospitalized patients and those with multiple comorbidities, including urinary tract infections, respiratory infections, and infections of the bones, blood, and central nervous system.^61^

Significant differences were found between patients with PCa and healthy patients, along with decreased levels of certain biochemical parameters, such as folic acid and biotin, in patients with PCa.^13^,^62^ In patients diagnosed with PCa, elevated activities of certain enzymes such as AST, ALT, and ALP are often observed. The increased activity of these enzymes may be related to various mechanisms, including liver cell damage, inflammation, hormonal treatment, and cancer metastasis, particularly in the liver and bone. We observed a significant decrease in liver enzymes, including ALT and AST, between the initial and final stages of the treatment (Figure 5). This reduction reflects the findings of other studies that have reported altered liver function in cancer patients, which can be attributed to both the direct effects of radiotherapy and changes in the gut microbiome. Elevated IL-6 levels have been implicated in the development of hormone resistance in PCa, further emphasizing the interconnectedness of systemic inflammation, cancer progression, and microbiome health. The enzyme ALP is particularly significant, as its elevated level is often associated with PCa metastasis to bones, which is a characteristic site for the spread of this cancer. In a study conducted by Zhou et al^63^ on recruited patients with PCa and benign prostatic hyperplasia (BPH), the mean AST and ALT values were 20.09 and 20.08 IU/L in PCa patients (N = 194), and the mean AST and ALT values in BPH patients were 19.37 and 21.71 IU/L (N = 210). Elevated cholesterol concentrations may be associated with tumorigenesis, because cholesterol is an essential component of cell membranes and may support the proliferation of cancer cells. Moreover, studies have suggested that high cholesterol levels may influence the development of resistance to hormonal therapy in PCa patients. Correlation analysis between fecal microbiota and urine and blood parameters provides additional insights into the role of the microbiome in modulating patient health. For instance, the strong correlation among *Streptococcus* levels, urine pH, and leukocyte levels highlights the potential systemic effects of gut dysbiosis (Figure 6). These observations underline the importance of maintaining gut microbiota balance during cancer treatment to mitigate adverse side effects and improve overall outcomes.

Research on the effects of ionizing radiation on bacterial communities shows inconsistent results.^64–66^ For instance, studies have reported conflicting trends in the abundance of *Bacteroidetes* and *Actinobacteria* during radiotherapy, while an increase in *Proteobacteria* is a more consistent finding.^64^ There is growing evidence that the gut microbiota influences PCa, for example, progression by affecting intestinal permeability.^67^,^68^ Increased permeability allows passive bacterial translocation through pathways involving tight junctions or transcellular mechanisms. Microorganisms such as *E. coli, Salmonella typhimurium, Clostridium perfringens* can produce toxins that damage intestinal barriers or alter permeability, facilitating translocation.^69–75^

The intestines harbor a unique and dynamic microbiome, constantly exposed to external stimuli including diet, infectious agents, antibiotics, and xenobiotics.^76–78^ These factors influence the microbiome composition of patients in ways not directly related to the treatment protocol. Diet, as a key determinant of microbiome composition, may have varied among participants, affecting substrate availability for bacteria and the overall stability of the gut ecosystem.^79–83^

Besides diet, medication intake is one of the most significant factors influencing changes in the gut microbiota.^84^ Studies by Imhann et al^85^ demonstrated that proton pump inhibitors (PPIs) and metformin affect gut microbiota composition. Other commonly used drugs, such as statins and antidepressants, are associated with distinct gut microbiota features.^85–89^ Consequently, this can influence the risk of developing intestinal infections or inflammatory bowel conditions and may also impact host metabolism.^90^,^91^ Finally, lifestyle factors, including physical activity levels, smoking, alcohol consumption, and stress, also play a role in shaping microbiome diversity and stability.^92^ In research conducted by Stewart et al^93^ tobacco smokers exhibited a higher relative abundance of *Prevotella*, a lower relative abundance of *Bacteroides*, and lower Shannon diversity compared to the control group. They also showed that tobacco smoking had a significant impact on bacterial profiles compared to e-cigarette users, with the most notable associations being an increased relative abundance of *Prevotella* (P = 0.006) and a decreased abundance of *Bacteroides* (P = 0.036) in the stool of tobacco smokers compared to e-cigarette users.

Finally, our findings highlight the need for further research on the role of the microbiome in PCa treatment. Future studies should focus on exploring therapeutic interventions, such as fecal microbiota transplantation (FMT), which may help restore microbiota balance and improve patient outcomes during and after radiotherapy. Moreover, the use of modern culturomic approaches, as demonstrated in our study, offers a promising path for identifying microbiota changes that are undetectable using traditional sequencing methods. Future research should also aim for a more comprehensive consideration of multiple additional variables to better analyze the impact of radiotherapy on the microbiome.

## Conclusion

Radiation therapy for PCa significantly alters the composition of the gut microbiota, as evidenced by a decreased abundance of bacteria such as *E. coli* and an increase in opportunistic pathogens, such as *R. kristinae* and *E. asburiae* after treatment. In our study, we observed differences in microbiota diversity in samples before and on the day of radiotherapy (K1 and K2) compared with the end of radiotherapy (K3), suggesting an effect of radio-remediation on microbiota numbers, which in turn translates into gut health. The strong correlation between changes in microbiota composition, leukocyte levels, and urine pH confirmed the link between intestinal dysbiosis and patient health parameters. Our results underscore the need for further research on interventions that restore the balance of microbiota, which can improve treatment outcomes and reduce the side effects of cancer therapies.

## Data Sharing Statement

All data generated or analyzed in this study are included in this published article (and its Supplementary Information Files). Raw data used for the analysis and drawing conclusions are available on Repository for Open Data RepOD at https://doi.org/10.18150/XP0Y5A.
